# Spatial Distribution of *Mycobacterium ulcerans* in Buruli Ulcer Lesions: Implications for Laboratory Diagnosis

**DOI:** 10.1371/journal.pntd.0004767

**Published:** 2016-06-02

**Authors:** Marie-Thérèse Ruf, Miriam Bolz, Moritz Vogel, Pierre F. Bayi, Martin W. Bratschi, Ghislain Emmanuel Sopho, Dorothy Yeboah-Manu, Alphonse Um Boock, Thomas Junghanss, Gerd Pluschke

**Affiliations:** 1 Swiss Tropical and Public Health Institute, Basel, Switzerland; 2 University of Basel, Basel, Switzerland; 3 Section Clinical Tropical Medicine, Heidelberg University Hospital, Heidelberg, Germany; 4 Fairmed, Bureau Régional pour l’Afrique, B.P. 5807, Yaoundé, Cameroon; 5 Centre de Depistage et de Traitement de l'Ulcere de Buruli d'Allada, Allada, Benin; 6 Noguchi Memorial Institute for Medical Research, University of Ghana, Legon, Ghana; Fondation Raoul Follereau, FRANCE

## Abstract

**Background:**

Current laboratory diagnosis of Buruli ulcer (BU) is based on microscopic detection of acid fast bacilli, quantitative real-time PCR (qPCR), histopathology or cultivation. Insertion sequence (IS) 2404 qPCR, the most sensitive method, is usually only available at reference laboratories. The only currently available point-of-care test, microscopic detection of acid fast bacilli (AFB), has limited sensitivity and specificity.

**Methodology/ Principal Findings:**

Here we analyzed AFB positive tissue samples (n = 83) for the presence, distribution and amount of AFB. AFB were nearly exclusively present in the subcutis with large extracellular clusters being most frequently (67%) found in plaque lesions. In ulcerative lesions small clusters and dispersed AFB were more common. Beside this, 151 swab samples from 37 BU patients were analyzed by IS2404 qPCR and ZN staining in parallel. The amount of *M*. *ulcerans* DNA in extracts from swabs correlated well with the probability of finding AFB in direct smear microscopy, with 56.1% of the samples being positive in both methods and 43.9% being positive only in qPCR. By analyzing three swabs per patient instead of one, the probability to have at least one positive swab increased from 80.2% to 97.1% for qPCR and from 45% to 66.1% for AFB smear examination.

**Conclusion / Significance:**

Our data show that *M*. *ulcerans* bacteria are primarily located in the subcutis of BU lesions, making the retrieval of the deep subcutis mandatory for examination of tissue samples for AFB. When laboratory diagnosis is based on the recommended less invasive collection of swab samples, analysis of three swabs from different areas of ulcerative lesions instead of one increases the sensitivity of both qPCR and of smear microscopy substantially.

## Author Summary

Currently, four laboratory methods are available to diagnose Buruli ulcer, a neglected tropical skin disease caused by *Mycobacterium ulcerans* affecting mainly children in remote rural areas of West Africa. Only one of the four methods, direct microscopic examination of wound exudate for acid fast bacilli, is suitable as point-of-care test. The others, histopathology, culture and IS2404 quantitative PCR, require sophisticated laboratory infrastructure. However, in comparison to the current gold standard, IS2404 quantitative PCR, microscopic smear examination has limited sensitivity. Our results on the distribution of *M*. *ulcerans* in Buruli ulcer lesions emphasize that the sensitivity of Buruli ulcer laboratory diagnosis is dependent on optimal sampling procedures. Accurate histopathology crucially depends on tissue samples containing all three skin layers, including the subcutis in which the majority of the bacteria are found. For IS2404 quantitative PCR, culture and direct smear detection, the margin of ulcerative lesions should be sampled at several positions, since bacteria and bacterial DNA are unevenly distributed. With optimized sampling, well-trained laboratory personnel and good microscopy infrastructure, direct smear examination reached a sensitivity of 73%, as compared to IS2404 quantitative PCR.

## Introduction

Buruli ulcer (BU) is a devastating disease of the skin and subcutis, resulting from infection with *Mycobacterium ulcerans* [1–3]. BU lesions present as non-ulcerative (papules, nodules, plaques or oedema) or ulcerative forms. Extensive ulcerated wounds often lead to permanent disabilities. Especially in remote rural areas of Africa diagnosis is often clinical and its accuracy depends strongly on the experience of the local health staff [4]. The differential diagnosis of BU includes diseases like cutaneous tuberculosis, cutaneous leishmaniasis and squamous cell carcinoma [3]. Because of this and also because the current standard antibiotic treatment is associated with relevant side effects it is important to reconfirm the initial clinical diagnosis by a laboratory test. The detection of the *M*. *ulcerans* specific insertion sequence 2404 (IS2404) in tissue samples, swabs or fine needle aspirates (FNA) by quantitative real-time PCR (qPCR) is the current gold standard to diagnose BU disease [3,4]. Other available diagnostic methods are based on the ability to culture the bacteria, the presence of typical histopathological features in skin biopsies or the direct detection of acid fast bacilli (AFB). Compared to qPCR, both microscopy and culture have limited sensitivity and may give false negative results [5]. This is in part due to the uneven distribution of the bacteria in BU lesions. Already in 1948 Mac Callum described the presence of “grouped masses of acid fast bacilli” inside BU lesions [6]. In 2006 Rondini *et al*. analyzed the horizontal distribution of AFB across entire lesions by correlating histopathology with semi-quantitative IS2404 qPCR results [7]. AFB were found to be focally clustered with some bacteria detectable in the peripheral, macroscopically healthy tissue [7].

For laboratory reconfirmation of clinically BU-suspicious ulcerative lesions it is recommended to swab the wounds with sterile cotton swabs [4]. Swabs can be used for culture, IS2404 qPCR analysis and for preparing smears for direct microscopic detection of AFB [4]. It is recommended by the World Health Organization (WHO) to swab the undermined edges instead of the lesion core because it is assumed that the bacterial load is higher there. The present study aimed at exploring the distribution and presence of AFB at the margin of ulcerative BU lesions and in plaques by qPCR, smear microscopy and histopathology in a large BU patient group.

## Materials and Methods

### Ethics statement

Ethical approval for analyzing patient specimens was obtained from the ethical review board of the Ministry of Health of Benin (N° IRB00006860), the Cameroon National Ethics Comitee (N°041/CNE/DNM/09,N°172/CNE/SE/2011 and ISRCTN72102977) and the Ghana Noguchi Memorial Institute for Medical research (FWA00001824). Written informed consent from the patients or their guardians was obtained before specimens were collected for reconfirmation of BU as well as for detailed histopathological analysis and all patient data have been anonymized.

### Tissue samples

Punch biopsies were taken before, during or after treatment, both for the initial diagnosis and subsequent monitoring of the response to treatment [8–11]. Excisions were only done for some patients, based on the judgment of the responsible clinician [8].

Altogether 378 tissue samples from 219 laboratory reconfirmed (at least one IS2404 qPCR positive swab) BU patients were histopathologically analyzed and AFB were found in 94 (24.9%) of these tissue samples coming from 72 patients. Eleven AFB positive skin samples had to be excluded from the analysis because of incomplete recovery of skin layers. The remaining 83 tissue specimens coming from 63 patients were analyzed for the abundance and distribution of AFB by histopathology. Lesions of 61/63 AFB positive patients were located at the extremities. 68/83 (81.9%) tissue samples came from ulcerative lesions and 15/83 (18.1%) from plaques. From ulcerated BU lesions punch biopsies were taken at the outer margin of the lesion at the border of the indurated to normal tissue, well beyond the undercutting to not miss the subcutis. In contrast, from BU plaque lesions punch biopsies were collected from the non-ulcerated center of the lesion.

### Histopathological analysis

Tissue samples (4 mm punch biopsies or surgical excisions) were aseptically removed and immediately fixed in 10% neutral-buffered formalin for 24 h to maintain tissue structures. Afterwards they were transferred to 70% ethanol for storage and transport. Tissue specimens were then dehydrated, embedded into paraffin, and cut into 5 μm thin formalin fixed paraffin embedded (FFPE) tissue sections. After deparaffinization and rehydration, sections were stained with Ziehl-Neelsen/Methylenblue (ZN, Sigma-Aldrich) according to WHO standard protocols [4].

Tissue sections were analyzed with a Leica DM2500 Microscope. Pictures were either taken with a Leica DFC 420C camera or with an Aperio ScanScope XT.

### IS2404 quantitative PCR

Sterile dry cotton swabs were used to swab roughly equal sectors along the circumference of the ulcers from 37 Cameroonian BU patients. From each patient 2–6 swabs (depending on the lesion size), altogether 151 swabs, were collected by moving in a clockwise manner along the borders of the lesions. Swabs were stored in the fridge until shipment to the Swiss TPH in Basel, Switzerland. DNA extraction of the whole swab and IS2404 qPCR was performed as described by Lavender *et al*. [12]. CT values of samples taken at the same day from the same lesion were compared with each other and the ∆CT value between the lowest and the highest CT value was determined. Based on the ∆CT value, lesions were categorized into three different groups: minimal heterogeneity (∆CT ≤ 5), medium heterogeneity (5 < ∆CT ≤ 10) and maximal heterogeneity (∆CT > 10). The ∆CT value reflects the differences in DNA amount and therefore the differences in bacterial load present at different positions of the lesions with a ∆CT of 3.32 correlating to 10 times more target DNA. For the qPCR runs 1/50 of the DNA extracted from the swabs was used.

### Direct microscopic detection of AFB

Before storage of the swabs for qPCR analysis, part of the wound material on each swab was applied onto a glass slide. Smears were fixed by pulling the glass slide three times through a flame. Glass slides were stained with ZN [4] and embedded into Eukitt (Sigma-Aldrich) mounting medium. Entire slides were analyzed for the presence of AFB with a Leica DM2500 microscope using the 40x and the 100x objectives.

### Statistical analysis

The proportion of positive qPCR or smear microscopy tests when different numbers of swabs are taken per patient was determined using a bootstrap with 1000 loops. 30 patients having had at least 4 swabs (134 swabs in total) were selected for the calculations. First, one swab per patient was chosen at random and the proportion of positive tests in the subsample was calculated. This was repeated 1000 times and the probability of obtaining one positive test when only one swab is taken was computed by taking the mean of all proportions. A similar procedure was followed to determine the probability of obtaining at least one or more positive tests when two, three or four swabs are taken form a patient.

## Results

### Preferential localization of *M*. *ulcerans* in the subcutis

**Fig 1:**
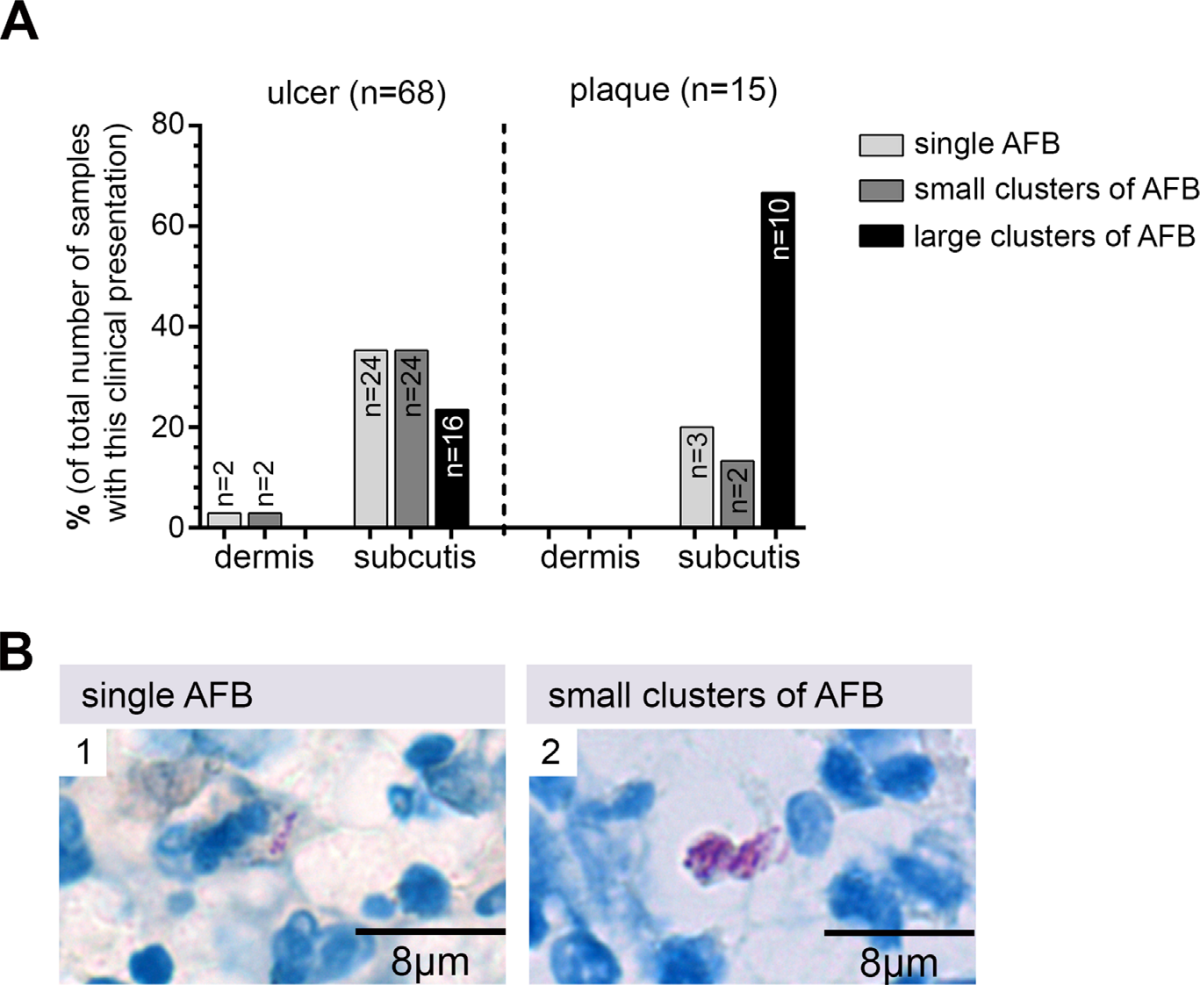
Preferential location of *M*. *ulcerans* bacteria in the subcutis. A: In plaque lesions mainly large clusters of extracellular bacteria were found, whereas in ulcerated lesions single AFB and small clusters were more common. In both types of lesions AFB were primarily found in the subcutis. B: Examples for dispersed single bacteria (1) and small clusters of AFB (2) in histological sections stained with ZN (counterstain methylenblue).

**Fig 2:**
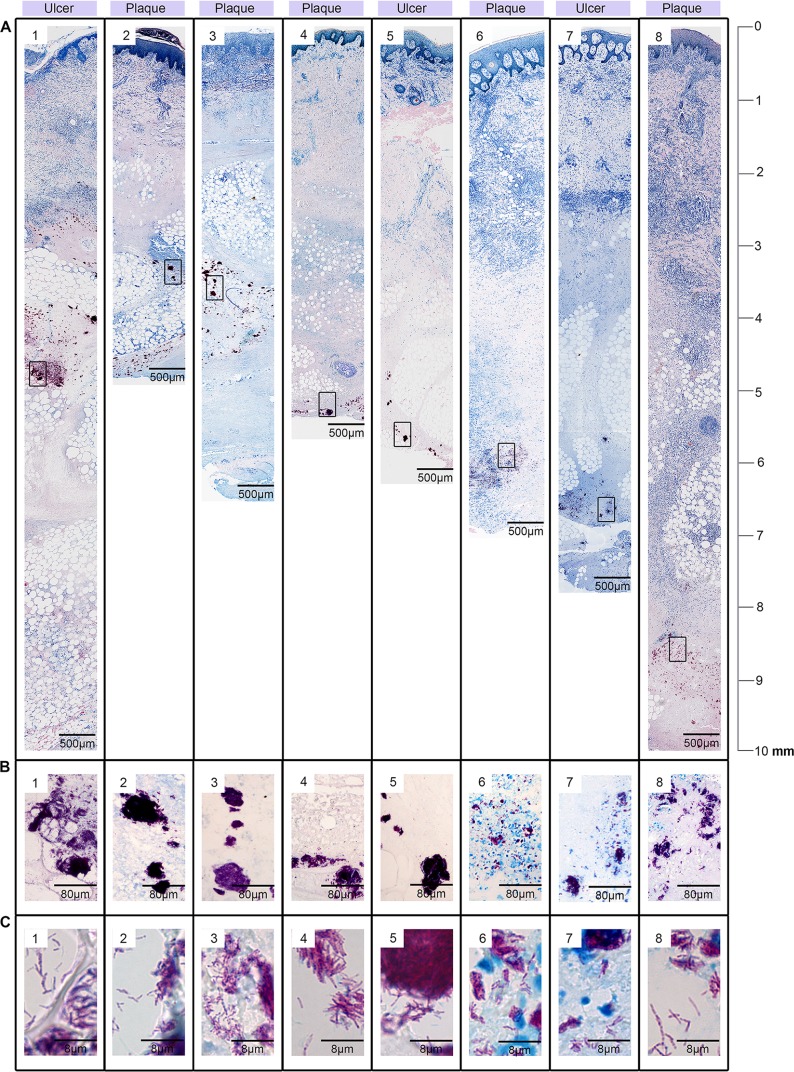
Localisation of large clusters of AFB in the subcutis of BU lesions. Histological sections were stained with ZN (counterstain methylenblue). A: Cross sections through the excised tissue specimens from BU plaque lesions (A2-A4, A7-A8) or ulcerated BU lesions (A1, A5-A6) revealing large clusters of AFB located in the subcutis in different tissue depths (3 mm—10 mm). Boxed areas are shown in a higher magnification in (B). B: Large clusters of AFB. C: High magnification of AFB with typical rod shaped appearance.

In histopathological sections of BU lesions AFB presented either as i. single bacteria (Fig 1B1), ii. small clusters of AFB (Fig 1B2) or iii. large clusters (Fig 2) located in a tissue depth of up to 10 mm in the FFPE sections (Fig 2). Since 10–30% shrinkage of the tissue is common in FFPE tissue sections the true skin depth of the *M*. *ulcerans* bacteria was up to 1.3 cm (in excisions). Similar numbers of tissue specimens could be assigned to each category: in 29/83 (35%) of the microscopy AFB positive specimens only single bacteria were found, 28/83 (34%) samples contained small clusters of AFB and 26/83 (31%) large clusters (Fig 1A). When specimens were further stratified by lesion type, 67% (10/15) of the analyzed plaque specimens presented with large clusters of extracellular bacteria, 13% (2/15) contained only small clusters and 20% (3/15) displayed only single bacteria. In contrast, single bacteria (26/68; 38%) and small clusters (26/68; 38%) dominated in the specimens originating from ulcers; here large clusters of AFB were found in only 16/68 (24%) of the samples (Fig 1A).

AFB were not evenly distributed across the three main skin layers, epidermis, dermis and subcutis. In none of the microscopy positive tissue samples AFB were detected in the epidermal layer. Similarly the upper part of the dermis was never infected by *M*. *ulcerans*. However, single cells or small clusters of AFB were occasionally found in the lower part of the dermis close to the subcutis. In all tissue specimens comprising also the subcutis (n = 83) the majority of AFB was found in this layer (Figs 1A and 2).

### Uneven distribution of *M*. *ulcerans* DNA at the margins of ulcerative lesions

**Fig 3:**
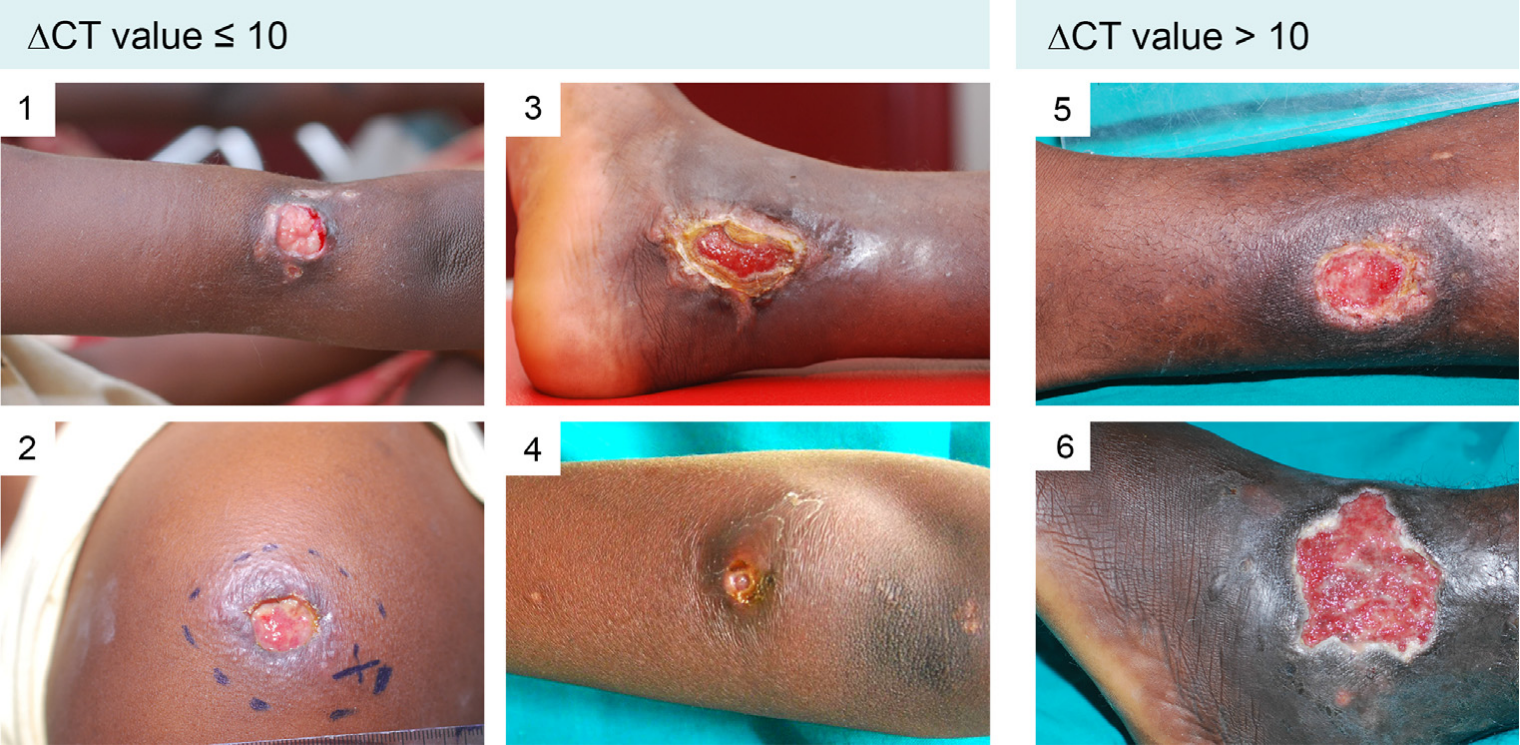
Examples of lesions presenting with both IS2404 qPCR positive and negative swabs. Lesions of six laboratory confirmed BU patients are shown, whereof four (# 1–4) presented with a ∆CT value ≤ 10, indicating a minimal to medium heterogeneity and two (# 5 and 6) presented with a ∆CT value > 10, indicative for a maximum heterogeneity. From each lesion four swabs have been taken in a clockwise manner and IS2404 qPCR has been performed.

We investigated the distribution of bacterial DNA and of AFB in ulcerative lesions of 37 untreated qPCR reconfirmed (at least one positive IS2404 qPCR positive swab; S1 and S2 Tables) BU patients by analyzing 151 swab samples taken from the lesion margins at different positions of the same lesions (Fig 3 and S1 and S2 Figs). Altogether 18.5% (28/151) of the total number of analyzed swabs were qPCR negative. The 28 qPCR negative swabs came from the lesions of 15 patients (15/37, 40%). A total of 66 swabs from these 15 patients have been analyzed (S1 Table) and 42.4% (28/66) of these swabs had a negative qPCR result. However, each of these 15 patients had at least one IS2404 qPCR positive swab. All 85 swabs taken from the other 22 patients were IS240 qPCR positive. In comparative qPCR analyses of different swabs taken from different areas of the same lesion, minimal heterogeneity (∆CT ≤ 5) was found in 9/37 (24%), medium heterogeneity (5 < ∆CT ≤ 10) in 17/37 (46%) and a maximal heterogeneity (∆CT > 10) in 11/37 (30%) of the lesions (S1 and S2 Figs and S1 and S2 Tables), where a ∆CT > 10 represents a more than 1000 fold difference in DNA quantity in different places of one lesion.

**Fig 4:**
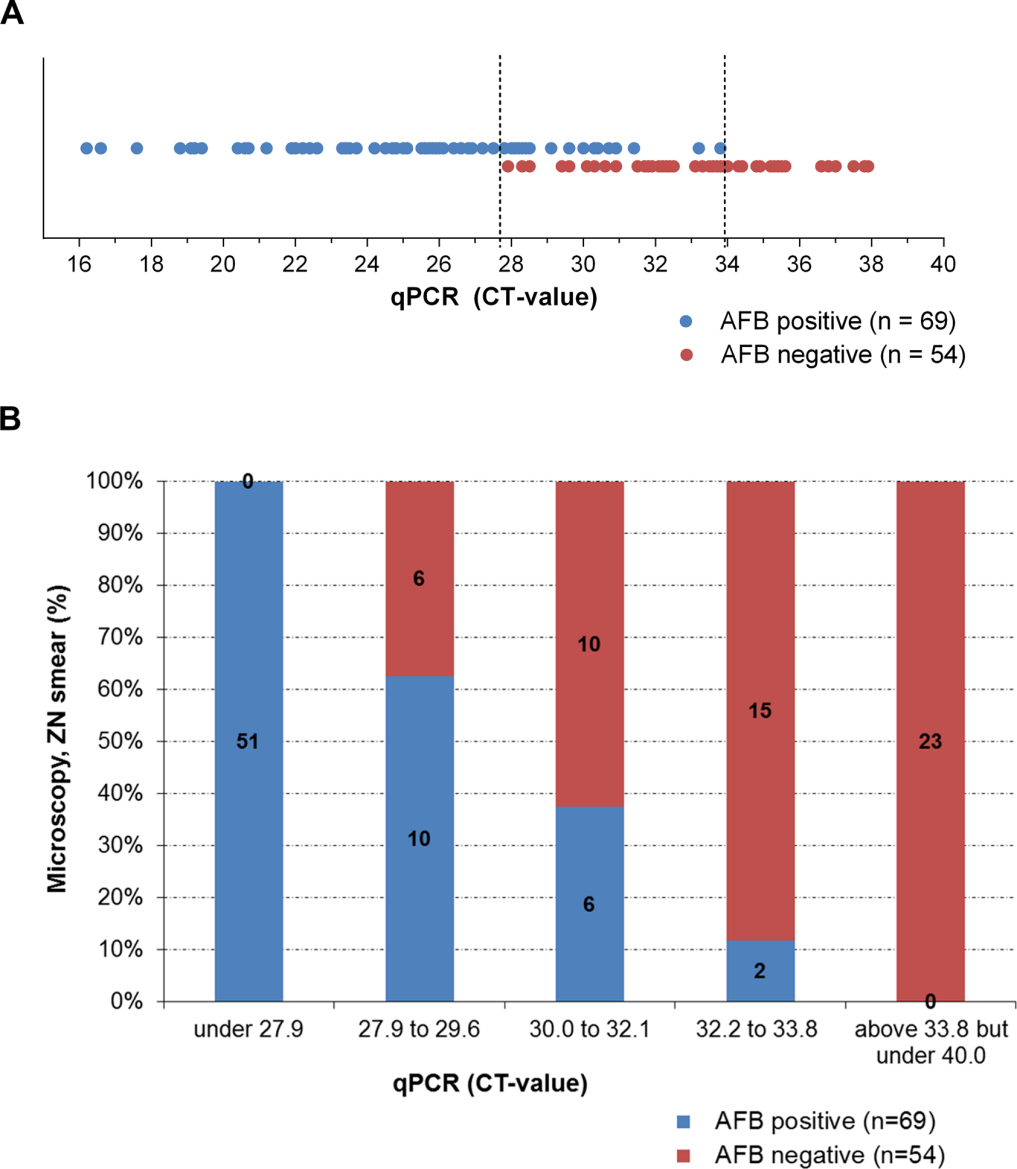
Correlation between direct smear microscopy and IS2404 qPCR results. Of the 123 IS2404 qPCR positive swab samples analyzed by direct smear microscopic analysis after ZN staining, 54 samples (43.9%) were only positive by qPCR, whereas 69 samples (56.1%) were positive for both methods applied. Up to a qPCR CT of 27.8 all swabs were positive for both methods. Between CT 27.9 and CT 33.8 results were variable and above CT 33.8 all samples were microscopy negative. A: AFB positive and negative swabs in correlation to the qPCR values. Each dot represents one swab sample. B: Percentage of AFB positive and negative swabs in correlation to qPCR value ranges.

The 151 swab samples, obtained from the 37 BU patients were also analyzed by microscopy after direct ZN staining (Fig 4 and S1 and S2 Tables). All 28 PCR negative swabs were also microscopy negative. Sixty-nine samples (56.1%) were positive in both tests and 54 samples (43.9%) were only qPCR positive (Fig 4). AFB were microscopically detected in smears of all 51 swabs (41.5%) yielding a qPCR CT value of ≤ 27.8 (equaling about ≥ 300 genomes in the total extract of the swab [13]). In contrast, AFB were found in none of the 23 swabs (18.7%) yielding a CT value ≥ 33.9 (equaling about ≤ 2 genomes in the total extract of the swab) [14]. Of the 49 swabs (39.8%) with a CT between 27.8 and CT 33.9, 18 (36.7%) were also positive in ZN smear microscopy (Fig 4A), with a clear increase in the proportion of positive ZN smears with decreasing qPCR CT value (Fig 4B). In total, 27/37 (73%) of the patients with at least one qPCR positive swab had also at least one positive microscopy result.

### Increase in test sensitivity by analyzing multiple swab samples

Results obtained with 134 swabs from those 30 patients who had at least four swabs taken from different lesion areas were statistically analyzed for the effect of testing multiple samples on assay sensitivity. Both for qPCR (Table 1) and for smear microscopy (Table 2) the probability of obtaining at least one positive test result per patient increased substantially, when analyzing two or three samples instead of one. In particular for qPCR the effect of analyzing a fourth swab was only marginal.

**Table 1:** Increase in the probability of obtaining one or more positive qPCR results with the number of swabs analyzed.

Number of swabs analyzed	Probability* of PCR positive tests (expressed as percentages)			
	% of one positive test (95% CI)	% of two positive tests (95% CI)	% of three positive test (95% CI)	% of four positive test (95% CI)
1	80.17 (79.81 to 80.52)			
2	92.16 (91.96 to 92.41)	67.67 (67.37 to 67.98)		
3	97.1 (96.25 to 97.24)	82.7 (82.47 to 82.95)	60.19 (59.95 to 60.43)	
4	98.69 (98.57 to 98.80)	92.01 (91.90 to 92.10)	73.33 (73.28 to 73.38)	55.82 (55.67 to 55.97)

* A bootstrap with 1000 loops was used

**Table 2:** Increase in the probability of obtaining one or more positive smear microscopy results with the number of swabs analyzed.

Number of swabs analyzed	Probability* of ZN smear positive tests (expressed as percentages)			
	% of one positive test (95% CI)	% of two positive tests (95% CI)	% of three positive test (95% CI)	% of four positive test (95% CI)
1	45.00 (44.63 to 45.38)			
2	59.01 (58.70 to 59.32)	30.67 (30.33 to 30.93)		
3	66.11 (65.87 to 66.35)	44.58 (44.37 to 44.79)	23.38 (23.14 to 23.61)	
4	71.26 (71.11 to 71.41)	51.30 (51.20 to 51.40)	38.01 (37.91 to 38.11)	18.52 (18.38 to 18.67)

* A bootstrap with 1000 loops was used

## Discussion

To date, no systematic analysis of the distribution of *M*. *ulcerans* bacteria in BU lesions has been published. Here we have systematically evaluated the amount and distribution of AFB in 83 tissue specimens in which we could detect AFB, corroborating previous findings that extracellular clusters of *M*. *ulcerans* bacteria in the subcutis are a histopathological hallmark of BU [6–8,14,15]. In none of the 83 analyzed samples we found AFB in the epidermal layer, whereas AFB were present in the subcutis of all complete tissue specimens with occasional minor spread of bacteria into the lower layer of the dermis. As expected [8], large extracellular clusters of AFB were found in most (67%) of the plaque lesions analyzed, but only in 24% of the ulcerated lesions, where small clusters and dispersed AFB were more common. This is most likely related to sloughing of necrotic tissue including the bulk of bacteria during ulcer formation [16,17]. Consequently, improper sampling of tissue specimens for histopathological analysis (e.g. not including the subcutis) will often result in false negative laboratory results based on microscopic detection of AFB in tissue specimens. Since no samples from nodular or edematous lesions were available we are not able to draw any conclusions concerning these pre-ulcerative forms.

Fine-needle aspiration (FNA) rather than taking punch biopsies is recommended [4] to obtain samples from clinically-diagnosed non-ulcerative lesions (nodules, plaques and oedema). However, FNA samples were not available for this study. Our findings on the distribution of AFB may reflect a tropism of *M*. *ulcerans* for the deeper subcutis, possibly related to special nutritional needs only provided by the destroyed fat tissue. However, it cannot be excluded that the location in the lower layers of the skin is rather the consequence of the inoculation route, which is still unclear, but may either be via skin trauma or by insect bites. Skin tropism is generally explained by the temperature preference of *M*. *ulcerans* [4,18,19].

In ulcerative BU lesions disease activity is thought to be concentrated at the edges of ulcers stretching into the indurated surroundings. Bacterial yield is expected to be highest below the undermined edges, where laboratory specimen for direct smear microscopy, qPCR and cultivation should be obtained. However, it needs to be emphasized that the textbook like presentation with circularly undermined edges is the exception rather than the rule in most confirmed BU lesions. In our patient cohort only a few patients had circular undermined edges, while all the others presented with only partial or even no undercutting (S1 and S2 Tables). Here we have addressed the question how evenly bacterial DNA is distributed in different areas of the lesion by taking several swabs from the same lesion but from different areas. Samples were analyzed in parallel by IS2404 qPCR and direct smear microscopy for AFB. A high variability in the distribution of *M*. *ulcerans* DNA in different samples of the same lesions was observed and from 40% of the lesions both IS2404 qPCR positive and negative swabs were obtained. This supports the concept to repeat sampling if qPCR results are negative, but clinical signs and symptoms are highly indicative for BU.

We observed a good correlation between the amount of *M*. *ulcerans* DNA in extracts from swabs and the positivity of direct smear microscopy. All swabs yielding extracts containing ≥ 300 *M*. *ulcerans* genomes had positive microscopy results, while swabs yielding ≤ 2 genomes were all negative. About 35% of the swabs with DNA amounts between the above limits were microscopy positive. While the sensitivity of direct AFB detection in smears was thus lower than that of IS2404 qPCR, 73% of all IS2404 qPCR positive patients were also confirmed by microscopy by analyzing several swabs per patient. This value is high compared to previous studies [20–22] and might not only be due to the number of swabs analyzed, but also to the covering of the slides with Eukitt mounting medium and a coverslip immediately after staining, which protects the slides from attachment of dust and dirt and good laboratory equipment. Our analyses also indicate that for qPCR analyzing three swab samples may be an optimal number. However we have to emphasize that for this study each swab was taken from a different area of the lesion and if the margin of the whole lesion is sampled with one swab, results may be different. Overall, all diagnostic tests rely on a careful sampling and in case of a negative result but a high clinical suspicion repeated testing may be advisable.

## Supporting Information

S1 Table Patients from whom not all analyzed swabs were positive by IS2404 qPCR. (DOCX)

S2 Table Patients from whom all analyzed swabs were positive by IS2404 qPCR. (DOCX)

S1 Fig Lesions from which both IS2404 qPCR positive and negative swabs were obtained. Depicted are all analyzed lesions that presented with both positive and negative IS2404 qPCR results, sorted by the ∆CT heterogeneity. Picture numbers correspond to the patient numbers in S1 Table.
(TIF)

S2 Fig Lesions from which only positive IS2404 qPCR swabs were obtained. All analyzed lesions that presented with positive IS2404 qPCR results, sorted by the ∆CT heterogeneity are shown. Picture numbers correspond to the patient numbers in S2 Table.
(TIF)
