# Supplementary material for: Spatial Distribution of Mycobacterium ulcerans in Buruli Ulcer Lesions: Implications for Laboratory Diagnosis
Source: PLoS Negl Trop Dis. 2016 Jun 2;10(6):e0004767. doi: 10.1371/journal.pntd.0004767 (PMC4890796; doi:10.1371/journal.pntd.0004767)
Supplement: S1 Table — (DOCX) [file pntd.0004767.s001.docx]

**Supplementary Table 1: Patients from whom not all analyzed swabs were positive by IS2404 qPCR**

| **Patient ID** | **Swab number** | **ZN (pos/neg)** | **qPCR**  **(CT value)** | **∆CT over all swabs** | **Ct value Heterogeneity**  **(Minimum, medium, maximum)** | **Number of PCR negative swabs** | **Lesion size**  **(cm^2^)** | **Undercutting**  **(none, partial, circular,** **ambiguous)** |
| --- | --- | --- | --- | --- | --- | --- | --- | --- |
| **1** | 1 | Neg | 40 | 4.8 | minimum | 4/5 | 1.9 | ambiguous |
|  | 2 | Neg | 40 |  |  |  |  |  |
|  | 3 | Neg | 40 |  |  |  |  |  |
|  | 4 | Neg | 35.2 |  |  |  |  |  |
|  | 5 | Neg | 40 |  |  |  |  |  |
| **2** | 1 | Neg | 32.3 | 7.7 | medium | 1/4 | 2 | partial |
|  | 2 | Neg | 35.4 |  |  |  |  |  |
|  | 3 | Neg | 32.4 |  |  |  |  |  |
|  | 4 | Neg | 40 |  |  |  |  |  |
| **3** | 1 | Neg | 30.9 | 9.1 | medium | 2/4 | 4.4 | none |
|  | 2 | Neg | 40 |  |  |  |  |  |
|  | 3 | Neg | 33.3 |  |  |  |  |  |
|  | 4 | Neg | 40 |  |  |  |  |  |
| **4** | 1 | Neg | 40 | 5.2 | medium | 3/5 | 1.1 | circular |
|  | 2 | Neg | 35.3 |  |  |  |  |  |
|  | 3 | Neg | 40 |  |  |  |  |  |
|  | 4 | Neg | 40 |  |  |  |  |  |
|  | 5 | Neg | 34.8 |  |  |  |  |  |
| **5** | 1 | Neg | 40 | 9.3 | medium | 4/5 | 29 | partial |
|  | 2 | Neg | 40 |  |  |  |  |  |
|  | 3 | Pos | 30.7 |  |  |  |  |  |
|  | 4 | Neg | 40 |  |  |  |  |  |
|  | 5 | Neg | 40 |  |  |  |  |  |
| **6** | 1 | Neg | 40 | 7.9 | medium | 2/4 | 1.3 | partial |
|  | 2 | Neg | 35.6 |  |  |  |  |  |
|  | 3 | Neg | 32.1 |  |  |  |  |  |
|  | 4 | Neg | 40 |  |  |  |  |  |
| **7** | 1 | Neg | 40 | 5.7 | medium | 2/4 | 0.3 | none |
|  | 2 | Neg | 37 |  |  |  |  |  |
|  | 3 | Neg | 34.3 |  |  |  |  |  |
|  | 4 | Neg | 40 |  |  |  |  |  |
| **8** | 1 | Pos | 33.2 | 6.8 | medium | 1/5 | 52 | none |
|  | 2 | Neg | 33.7 |  |  |  |  |  |
|  | 3 | Neg | 34 |  |  |  |  |  |
|  | 4 | Neg | 37.5 |  |  |  |  |  |
|  | 5 | Neg | 40 |  |  |  |  |  |
| **9** | 1 | Neg | 34 | 11.7 | medium | 2/4 | 7 | none |
|  | 2 | Neg | 28.3 |  |  |  |  |  |
|  | 3 | Neg | 40 |  |  |  |  |  |
|  | 4 | Neg | 40 |  |  |  |  |  |
| **10** | 1 | Neg | 40 | 15 | maximum | 1/3 | 7.9 | none |
|  | 2 | Neg | 35.3 |  |  |  |  |  |
|  | 3 | Pos | 25 |  |  |  |  |  |
| **11** | 1 | Pos | 26.1 | 13.9 | maximum | 1/4 | 2.1 | partial |
|  | 2 | Pos | 27.5 |  |  |  |  |  |
|  | 3 | Pos | 30 |  |  |  |  |  |
|  | 4 | Neg | 40 |  |  |  |  |  |
| **12** | 1 | Pos | 28 | 14 | maximum | 1/6 | N.D. | partial |
|  | 2 | Neg | 32.2 |  |  |  |  |  |
|  | 3 | Neg | 33.8 |  |  |  |  |  |
|  | 4 | Neg | 35.5 |  |  |  |  |  |
|  | 5 | Neg | 40 |  |  |  |  |  |
|  | 6 | Pos | 26 |  |  |  |  |  |
| **13** | 1 | Neg | 40 | 14.4 | maximum | 1/5 | 22 | partial |
|  | 2 | Neg | 29.6 |  |  |  |  |  |
|  | 3 | Pos | 25.6 |  |  |  |  |  |
|  | 4 | Neg | 37.9 |  |  |  |  |  |
|  | 5 | Neg | 34.3 |  |  |  |  |  |
| **14** | 1 | Neg | 34 | 14.3 | maximum | 1/4 | 5.3 | none |
|  | 2 | Pos | 25.7 |  |  |  |  |  |
|  | 3 | Neg | 40 |  |  |  |  |  |
|  | 4 | Neg | 33.7 |  |  |  |  |  |
| **15** | 1 | Neg | 40 | 13.9 | medium | 2/4 | 21 | partial |
|  | 2 | Neg | 36.6 |  |  |  |  |  |
|  | 3 | Neg | 40 |  |  |  |  |  |
|  | 4 | Pos | 26.1 |  |  |  |  |  |
