# Supplementary material for: Spatial Distribution of Mycobacterium ulcerans in Buruli Ulcer Lesions: Implications for Laboratory Diagnosis
Source: PLoS Negl Trop Dis. 2016 Jun 2;10(6):e0004767. doi: 10.1371/journal.pntd.0004767 (PMC4890796; doi:10.1371/journal.pntd.0004767)
Supplement: S2 Table — (DOCX) [file pntd.0004767.s002.docx]

**Supplementary Table 2: Patients from whom all analyzed swabs were positive by IS2404 qPCR**

| **Patient ID** | **Swab number** | **ZN (pos/neg)** | **qPCR**  **(CT value)** | **∆CT over all swabs** | **Ct value Heterogeneity**  **(Minimum, medium, maximum)** | **Number of PCR negative swabs** | **Lesion size**  **(cm^2^)** | **Undercutting**  **(none, partial, circular, ambiguous)** |
| --- | --- | --- | --- | --- | --- | --- | --- | --- |
| **1** | 1 | Neg | 33.1 | 0.7 | minimum | 0/3 | 1.2 | ambiguous |
|  | 2 | Neg | 33.5 |  |  |  |  |  |
|  | 3 | Neg | 32.4 |  |  |  |  |  |
| **2** | 1 | Pos | 22 | 2 | minimum | 0/4 | 0.3 | circular |
|  | 2 | Pos | 22.4 |  |  |  |  |  |
|  | 3 | Pos | 20.4 |  |  |  |  |  |
|  | 4 | Pos | 20.7 |  |  |  |  |  |
| **3** | 1 | Neg | 32.3 | 0.9 | minimum | 0/2 | 2.1 | none |
|  | 2 | Pos | 31.4 |  |  |  |  |  |
| **4** | 1 | Pos | 23.5 | 3.4 | minimum | 0/3 | 0.5 | none |
|  | 2 | Pos | 26.9 |  |  |  |  |  |
|  | 3 | Pos | 24.8 |  |  |  |  |  |
| **5** | 1 | Pos | 28.4 | 3.2 | minimum | 0/5 | 8.5 | none |
|  | 2 | Pos | 29.1 |  |  |  |  |  |
|  | 3 | Pos | 26.4 |  |  |  |  |  |
|  | 4 | Pos | 29.6 |  |  |  |  |  |
|  | 5 | Neg | 28.5 |  |  |  |  |  |
| **6** | 1 | Pos | 17.6 | 1.5 | minimum | 0/2 | N.D. | partial |
|  | 2 | Pos | 19.1 |  |  |  |  |  |
| **7** | 1 | Neg | 33.6 | 0.3 | minimum | 0/2 | 1.8 | none |
|  | 2 | Neg | 33.3 |  |  |  |  |  |
| **8** | 1 | Pos | 30.9 | 2.6 | minimum | 0/2 | 0.7 | none |
|  | 2 | Pos | 28.3 |  |  |  |  |  |
| **9** | 1 | Pos | 28.1 | 6.6 | medium | 0/5 | 1.7 | none |
|  | 2 | Pos | 25.9 |  |  |  |  |  |
|  | 3 | Pos | 28.2 |  |  |  |  |  |
|  | 4 | Neg | 30.1 |  |  |  |  |  |
|  | 5 | Neg | 32.5 |  |  |  |  |  |
| **10** | 1 | Pos | 24.8 | 9.6 | medium | 0/5 | 14 | partial |
|  | 2 | Pos | 33.8 |  |  |  |  |  |
|  | 3 | Pos | 27.8 |  |  |  |  |  |
|  | 4 | Pos | 24.2 |  |  |  |  |  |
|  | 5 | Pos | 25.5 |  |  |  |  |  |
| **11** | 1 | Pos | 25.8 | 7 | medium | 0/5 | 10 | partial |
|  | 2 | Pos | 19.4 |  |  |  |  |  |
|  | 3 | Pos | 22.2 |  |  |  |  |  |
|  | 4 | Pos | 18.8 |  |  |  |  |  |
|  | 5 | Pos | 21.9 |  |  |  |  |  |
| **12** | 1 | Pos | 27.2 | 8.5 | medium | 0/4 | 9 | partial |
|  | 2 | Neg | 31.9 |  |  |  |  |  |
|  | 3 | Neg | 34.4 |  |  |  |  |  |
|  | 4 | Pos | 25.9 |  |  |  |  |  |
| **13** | 1 | Pos | 22.4 | 9.3 | medium | 0/4 | 0.5 | ambiguous |
|  | 2 | Pos | 24.7 |  |  |  |  |  |
|  | 3 | Pos | 30.3 |  |  |  |  |  |
|  | 4 | Neg | 31.7 |  |  |  |  |  |
| **14** | 1 | Pos | 26.8 | 6.1 | medium | 0/4 | 0.6 | circular |
|  | 2 | Pos | 26.9 |  |  |  |  |  |
|  | 3 | Neg | 30.6 |  |  |  |  |  |
|  | 4 | Pos | 24.5 |  |  |  |  |  |
| **15** | 1 | Neg | 31.5 | 6.3 | medium | 0/5 | 2.8 | partial |
|  | 2 | Neg | 33.9 |  |  |  |  |  |
|  | 3 | Neg | 32.4 |  |  |  |  |  |
|  | 4 | Neg | 36.8 |  |  |  |  |  |
|  | 5 | Neg | 37.8 |  |  |  |  |  |
| **16** | 1 | Neg | 27.9 | 6.2 | medium | 0/4 | 12 | partial |
|  | 2 | Pos | 25.6 |  |  |  |  |  |
|  | 3 | Neg | 31.8 |  |  |  |  |  |
|  | 4 | Neg | 29.6 |  |  |  |  |  |
| **17** | 1 | Pos | 30.4 | 7.8 | medium | 0/4 | 6.2 | none |
|  | 2 | Pos | 22.6 |  |  |  |  |  |
|  | 3 | Pos | 23.4 |  |  |  |  |  |
|  | 4 | Pos | 26.6 |  |  |  |  |  |
| **18** | 1 | Pos | 23.7 | 14.1 | maximum | 0/5 | 10.5 | partial |
|  | 2 | Neg | 35.3 |  |  |  |  |  |
|  | 3 | Pos | 23.4 |  |  |  |  |  |
|  | 4 | Pos | 22.2 |  |  |  |  |  |
|  | 5 | Pos | 21.2 |  |  |  |  |  |
| **19** | 1 | Pos | 23.5 | 14.3 | maximum | 0/5 | 2.5 | partial |
|  | 2 | Pos | 20.6 |  |  |  |  |  |
|  | 3 | Pos | 23.3 |  |  |  |  |  |
|  | 4 | Pos | 25.1 |  |  |  |  |  |
|  | 5 | Neg | 34.9 |  |  |  |  |  |
| **20** | 1 | Pos | 28.4 | 12.2 | maximum | 0/4 | 10.4 | partial |
|  | 2 | Pos | 16.6 |  |  |  |  |  |
|  | 3 | Pos | 19.2 |  |  |  |  |  |
|  | 4 | Pos | 27.2 |  |  |  |  |  |
| **21** | 1 | Pos | 28.5 | 11 | maximum | 0/4 | 59 | partial |
|  | 2 | Neg | 30.3 |  |  |  |  |  |
|  | 3 | Pos | 23.4 |  |  |  |  |  |
|  | 4 | Neg | 34.4 |  |  |  |  |  |
| **22** | 1 | Pos | 16.2 | 15.7 | maximum | 0/4 | 4.7 | partial |
|  | 2 | Pos | 28 |  |  |  |  |  |
|  | 3 | Neg | 29.4 |  |  |  |  |  |
|  | 4 | Neg | 31.9 |  |  |  |  |  |
