# Supplementary figures and images for: Spatial Distribution of Mycobacterium ulcerans in Buruli Ulcer Lesions: Implications for Laboratory Diagnosis
Source: PLoS Negl Trop Dis. 2016 Jun 2;10(6):e0004767. doi: 10.1371/journal.pntd.0004767 (PMC4890796; doi:10.1371/journal.pntd.0004767)

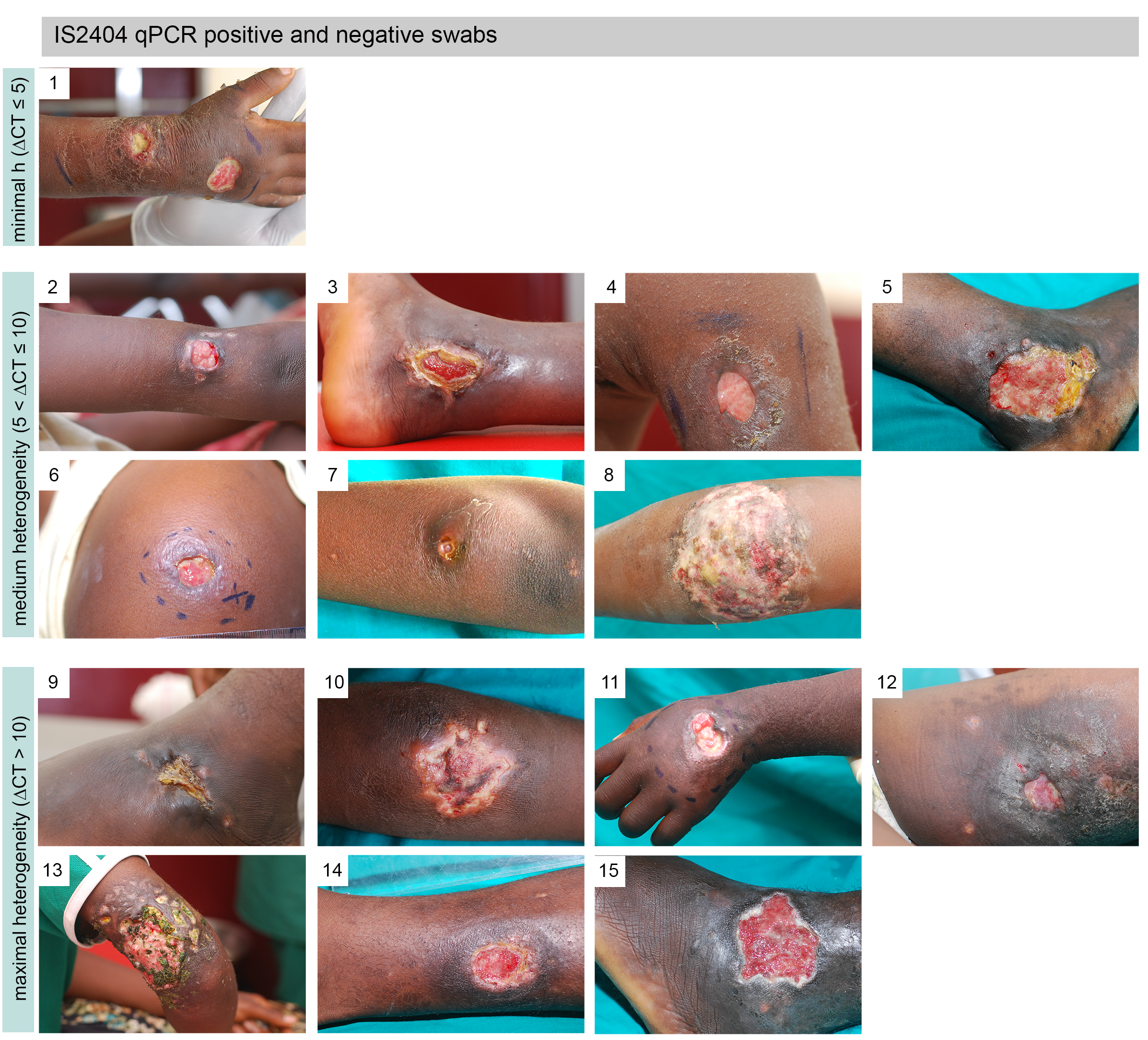

Supplement: S1 Fig — Depicted are all analyzed lesions that presented with both positive and negative IS2404 qPCR results, sorted by the ∆CT heterogeneity. Picture numbers correspond to the patient numbers in S1 Table. (TIF) [file pntd.0004767.s003.tif]

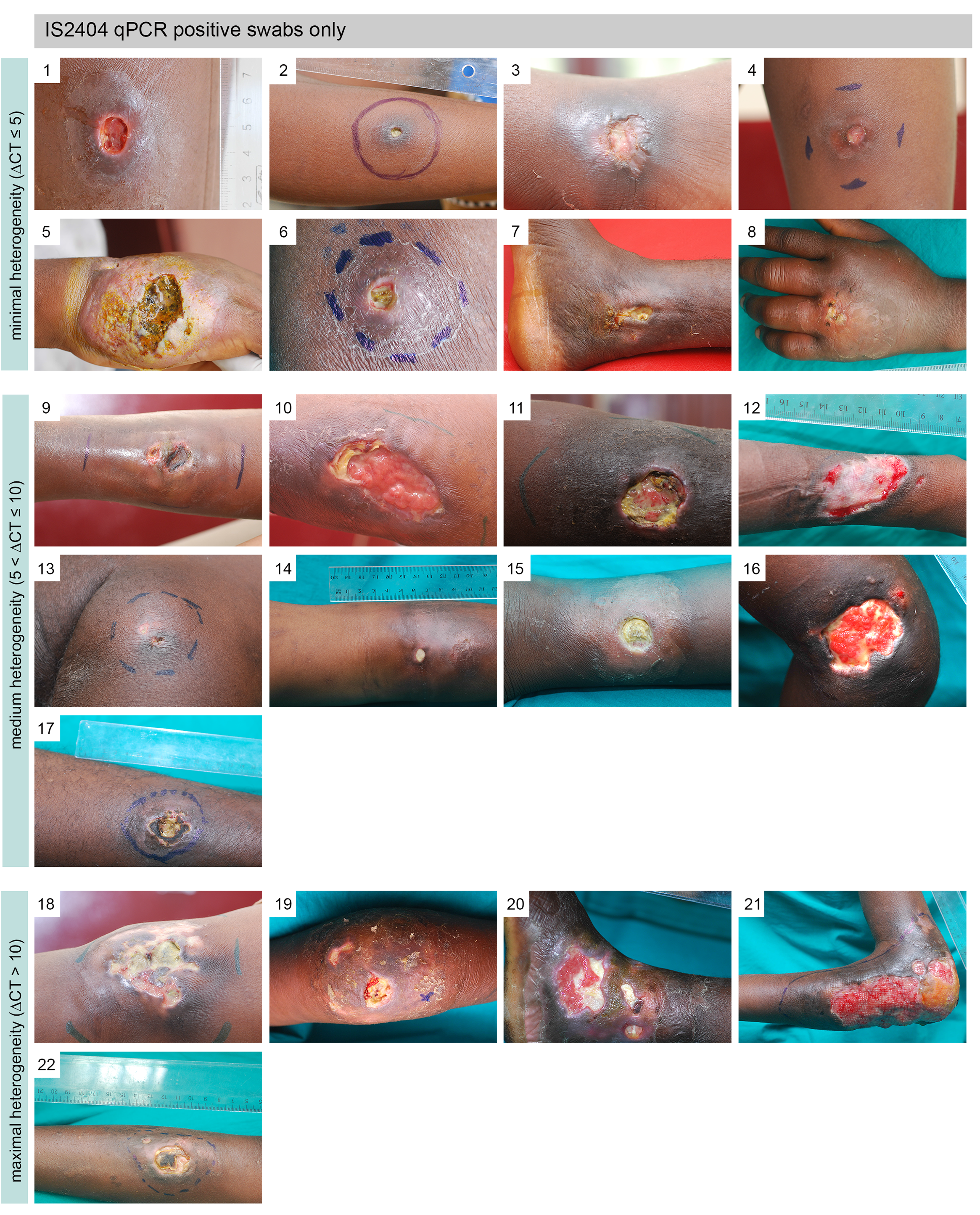

Supplement: S2 Fig — All analyzed lesions that presented with positive IS2404 qPCR results, sorted by the ∆CT heterogeneity are shown. Picture numbers correspond to the patient numbers in S2 Table. (TIF) [file pntd.0004767.s004.tif]
